# Innovative digital technology adapted in nursing education between Eastern and Western countries: a mini-review

**DOI:** 10.3389/fpubh.2023.1167752

**Published:** 2023-05-24

**Authors:** Mei-Chin Wang, Jing-Shia Tang, Yueh-Ping Liu, Chia-Chang Chuang, Chung-Liang Shih

**Affiliations:** ^1^Department of Nursing, Chung Hwa University of Medical Technology, Tainan, Taiwan; ^2^Department of Emergency Medicine, National Cheng Kung University Hospital, College of Medicine, National Cheng Kung University, Tainan, Taiwan; ^3^Department of Medical Affairs, Ministry of Health and Welfare, Taipei, Taiwan; ^4^Department of Emergency Medicine, College of Medicine, National Taiwan University, Taipei, Taiwan

**Keywords:** digital technology, e-learning, podcasts, nursing education, mobile learning

## Abstract

Advanced digital technologies have overcome the limitation of on-site teaching, especially after the COVID-19 epidemic. Various newly-developed digital technologies, such as e-learning, virtual reality, serious games, and podcasts, have gained renewed interest and come into the spotlight. Podcasts are becoming increasingly popular in nursing education as they provide a convenient and cost-effective way for students to access educational content. This mini-review article provides an overview of the development of podcasts in nursing education in Eastern and Western countries. It explores potential future trends in the use of this technology. The literature review demonstrates that nursing education in Western countries has already integrated podcasts into curriculum design, using the podcast to convey nursing education knowledge and skills and to improve students’ learning outcomes. However, few articles address nursing education in Eastern countries. The benefits of integrating podcasts into nursing education appear far greater than the limitations. In the future, the application of podcasts can serve not only as a supplement to instructional methodologies but also as a tool for clinical practicing students in nursing education. In addition, with the aging population increasing in both Eastern and Western countries, podcasts have the potential to serve as an effective delivery modality for health education in the future, particularly for the older adult, whose eyesight declines with age, and those populations with visual impairments.

## 1. Introduction

In recent years, rapid technological change has driven innovation in teaching strategies. Educators have combined digital technology with teaching methods and administrative strategies in ways that suit course goals, settings, and subject matter. With the rise of the COVID-19 pandemic, many schools had to conduct distance education, over long periods and in repeated intermittent waves, in response to the unpredictable occurrence of new outbreaks. Several countries have adopted educational strategies that integrate newly-developed technology devices into courses, e.g., e-learning (1, 2), augmented reality (3, 4), virtual reality (5, 6), mixed reality (7, 8), educational chatbot (9, 10) or podcasting (11–14). Newly-developed technology was used, springing up throughout the field of education. These devices have overcome many challenges throughout the education field, especially nursing education. Nursing education courses implement many skills by students’ hands and training how to achieve critical thinking. Of these technologies, podcasts have emerged as a more easily accessible and engaging tool for supplementing educational content. Recently, they have become increasingly important learning tools (15, 16). This mini-review article aims to overview the application of podcast technology and its current state in nursing education, which may shed light on possible future trends.

### 1.1. The origins and development of podcast

Podcasts start back to the 1980s. At that time, digital technology was booming, leading radio to evolve into new, far-reaching forms. The term “podcast” is derived from “iPod” combined with “broadcast.” The term *podcast* was first coined by the journalist Ben Hammersly from The Guardian in a newspaper article in 2004 (17). The definition of a podcast is an audio frequency launching via the internet, a platform homepage, or certain online portals, e. g., iTunes, or YouTube (17). With the advent of social media and the cloud, podcasting featured a decentralized open architecture in which audio content is stored on the website and allows users to link and download via RSS, aka. “Rich Site Summary” (or “Real Simple Syndication”). RSS was first developed by Dan Libby and Ramanathan V. Guha at Netscape in 1999, and by adding the open RSS into Apple’s platform, the podcast was transformed into a digital medium for mass consumption with the first business models stemming from the United States since 2012 (“the second age of podcasting”) (18). The podcast is soon becoming popular in Western countries because of its convenient properties, such as fast delivery, lower cost, and exceptional user-friendliness (15, 19). In addition to independent and amateur users, podcasts are also applied by educators for knowledge exchanges, e.g., the University of Oxford provided 254 free podcasts of entire courses and lessons on iTunes in 2013. Moreover, podcasts played an integral role in the continuing education development in emergency medicine and critical care (18, 20). In Eastern countries, such as Taiwan, podcasting did not gain a high profile until 2020 when two local companies– SoundOn and Firstory– provided a high-quality Chinese interface with convenient access technology via smartphones, Bluetooth, and an internet discount package to create a suitable podcast development ecosystem (21). In 2000, Taiwan only had 300 podcasts in the market, but it increased to more than 870 new podcasts in the first half of 2020. Furthermore, the frequency of downloads in Taiwan’s podcasts in December 2020 was 446 times and even 579 times in January 2021 compared to Jan. 2020 (22). Currently, podcast programs have grown drastically and steadily month-to-month by about 67% in Taiwan since 2020. Taiwanese’s favorite podcast programs are related to *entertainment*, *interests*, and *professional knowledge*, subsequently. *Society/culture*, *news/politics*, and *gossip* topics were the general interests.

In general, using podcasts can benefit educational pursuits. For example, podcasts reduced visual fatigue levels, enabled cyclical listening to heighten learning effectiveness, and improved the learning experience. According to the 2020 Edison Research survey, more than half of American audiences over 12 have the habit of listening to podcasts (23). Sixty percent of Taiwanese podcast listeners are of the average age of 23–32, among which nearly 95% have a college degree or above, and more than half of them have the habit of listening to podcasts (24). In Taiwan, the government promoted distance learning in higher education in the early 1990s (25). Taiwan is a global front-runner in digital technology development. Taiwanese nursing educators continually work with new technologies and innovations to apply various high-technology devices in nursing curriculum teaching strategies. However, podcasts were rarely used in healthcare-related education in Taiwan.

## 2. Method

Five major electronic databases, including PubMed database (MEDLINE), EBSCO Essentials (EBSCO), EMBASE (EMB), and Scopus, from 2008 to March 2023, were compiled in our review. All articles were from the Science Citation Index (SCI) and Social Science Citation Index (SSCI) databases. Upon conducting a thorough literature review, we extracted relevant keywords with a high frequency of occurrence to be used as search queries. These keywords include: ‘Podcast,’ ‘podcasting,’ ‘webcasts as a topic,’; ‘mobile learning,’ ‘digital storytelling,’ ‘e-learning,’; ‘nursing students,’ ‘baccalaureate nursing students,’; ‘nursing undergraduates,’ ‘midwifery,’ ‘nursing,’ ‘students’; ‘nursing education,’ ‘education,’ ‘nursing,’ all within the context of nursing education. Similar articles included in references were also screened. The inclusion criteria were reports or peer-reviewed studies of nursing education and training programs; peer-reviewed studies, accepted articles for publication, e.g., electronic publications (Epubs), and proceedings written in English. The exclusion criteria were conference abstracts, unpublished manuscripts, and whitepapers available online; articles not published in English.

## 3. Results

As we have already mentioned in Methodology, a total of 104 relevant articles was screened from the database: 32 from PubMed, 20 from EBSCO, 14 from EMB, and 38 from Scopus. After duplicates were removed, only 34 articles were assessed for eligibility.

### 3.1. Current literature reviews of podcasts applied in nursing education

Although podcasts were widely applied in medical education 20 years ago (20), the nursing education field only began to include podcasts in 2006 (26). According to the literature of synthesis evidence, 242 articles from a comprehensive literature database search related to the use of podcasts in nursing education were screened to identify 26 articles distinctly associated with the use of podcasts in nursing or midwife education. Podcasting was applied to a wide range of course topics (27), including basic medical courses, such as pharmacology (28), pathophysiology (29), microbiology or biology (16); professional knowledge in the nursing field such as evidence-based care research (30), donor egg recipients (31), training critical thinking or reflective thinking (32), The integrative review article confirmed that nursing educational material in podcasts as an assistance learning instrument applied to train nurses and midwives seemed particularly effective for learning new knowledge and skills (27).

In contrast to its limited use in nursing education, the podcast has been effectively applied to English and Chinese language learning courses in Taiwanese education (33–35). In nursing education, there are a few known instances of Taiwanese nursing educators using podcasts for master’s courses. In these cases, the master’s students were preceptors who were trained to design clinical nursing practice related to issues from the podcast contents. This process was followed up with discussion, reflection, and educational strategies for improving the preceptors’ instruction for beginner-level educational strategies. Hence, nursing educators are mulling over podcasts from social media, which can be regarded as a kind of nursing education material, especially for courses that must be committed to memory, require deep comprehension, skillful step-by-step clinical ability, and training in critical thinking (32, 36, 37).

### 3.2. The benefits and negative impact of the podcast application in nursing education

During COVID-19, podcasting brought convenience and entertainment to people worldwide while gaining popularity as a useful mobile digital device. In the past, the application and discussion of podcasting in nursing education were relatively unclear regarding its benefits and negative impacts. However, conducting a literature review regarding the use of podcasting in nursing education elicited its positive and negative effects, as outlined below.

### 3.3. The positive impact of the podcast on nursing education

#### 3.3.1. To obtain specialized knowledge and effectiveness of learning

Previous studies showed that courses using podcasts were designed to maximize their advantages, including the opportunity to listen repeatedly to the contents of the subject matter for better understanding (38). Most nursing students agreed that specialized basic medical knowledge could strengthen memory via listening to podcasting. Hence, raising learned effectiveness through podcasts in the nursing education field is a highly recommended teaching strategy.

#### 3.3.2. To raise comprehension and improve proficient clinical skills

Listening to podcasting helped students review clinical skills and knowledge during examination preparation, such as newborn infant physical examination (37). Results showed that most students preferred professional courses that blended podcasting, which could be accurately and consistently used to deliver information for each student (37). In addition, helping students pay attention to omitted issues in the lecture and revise error items of the examination through podcasting (37, 39). Other studies found that students reported better understanding by repeatedly listening to course content through podcasts (19, 29, 38), and about 83% of students use podcasts to help revise course content and enhance learning (16, 39). Therefore, podcasts were integrated into the curriculum, and through continued listening and repeated practice, students’ comprehension could be greatly improved, and further students’ motivation to learn was initiated (19).

#### 3.3.3. To improve self-confidence and communication skills

Nursing education integrated podcasts into lectures, which not only enhanced the self-confidence of nursing students but also improved the students’ communication skills (40, 41). When students had a strong self-efficacy for learning, their learning motivation was greatly improved (42). Another study showed that digital storytelling strategy design was applied to nursing students’ clinical practice via a podcasting device; students acquired other perspectives of care experience and recipients’ requirements, which furthered their learning of how to apply the concept of care for patients in the same situation (40, 43). More than 80% of students were willing to accept its course learning different experiences to deal with clinical issues (40). Other study results showed that nursing students positively apprised the use of podcasts to promote their knowledge and confidence about delirium awareness, and 96.32% of nursing students deemed that the podcast met their learning needs about delirium (44). Two studies also used podcasting to deliver palliative care and health information concepts (43, 45). Demonstrate that nursing philosophy knowledge can be released to students through broadcasts not limited to lectures. However, in nursing core competencies such as caring concepts, teamwork communication skills, and critical thinking are essential professional core competencies for nursing students, where podcasting as an emerging educational tool can assist students in this essential core competency learning (46).

#### 3.3.4. To provide a useful tool for lectures revision and strengthen supplementary perception

Other studies found that podcasts benefit students as a tool for review in the education field (16, 28, 46, 47) and provide instructor diversity in learning strategies and supplementary material (48). Furthermore, other studies found that podcasting was practical and convenient when students needed to shift places or take transportation (29, 31, 36), and most students deemed podcasting a useful learning tool (16, 28). Through the literature reviews, the advantages of podcasting have been summarized in Table 1.

**Table 1:** Thematic extraction from podcast applications in nursing education.

Study	Purpose	Methods	Results
Theme 1: To obtain specialized knowledge and effectiveness of learning			
Abate (36)/United States	Evaluation of the effectiveness of pharmacology courses by podcasts promoted knowledge retention and application in nursing students.	Design: This pilot study with a randomized controlled study	Students of the segmented podcast lecture group showed higher scores on multiple-choice and case-study assessments than those in the other two groups.
		Participants: A convenience sample of 35 female undergraduate nursing students were randomly assigned to one of three groups: traditional lecture (*n* = 12), unsegmented podcast lecture (*n* = 11), or segmented podcast lecture (*n* = 12).	
		Intervention: The face-to-face lecture was about 90 min. The nonstop podcast lecture was just over 57 min. The segmented podcast lecture, just over 51 min long, consisted of three sections. Podcasting by computer or MP3.	
Mostyn et al. (16)/United States	This study was to explore nursing students’ perceptions of the usefulness of supplementary biology podcasts.	Design: A mixed methods study	Students reported podcasts aided revision and helped promote understanding of course content 83 and 72%, respectively from the focus group participants who discussed finding podcasts especially useful in terms of revision.
		Participants: The first-year diploma/BSc nursing programmed students (*n* = 153) received Biological science podcasts supplementary learning tools and conducted two focus groups interview (*n* = 6)	
		Intervention: Nine live biological science lectures were recorded by staff on each of the university teaching sites and podcasts were made available to students across both sites via WebCT.	
Strickland et al. (30)/United Kingdom	Evaluation of the effectiveness of evidence-based nursing linked between the theoretical content and research in practice through podcasts.	Design: Not explicitly stated.	Total 77% of participants that they were easy to understand course content through podcast learning and increased student/tutor relationships leading to greater engagement.
		Participants: A convenience sample of two cohorts of students (cohort 1, *n* = 228; cohort 2, *n* = 233) from the Research and Evidence-Based Practice module were asked to evaluate the use of podcasts as part of the module evaluation process.	
		Intervention: A series of five podcasts linked to the podcasting host service at appropriate times within the module content in WebCT. The students could subscribe to the podcast feed and have these podcasts delivered automatically to their mobile devices	
Abedian et al. (31)/Iran	Evaluation of the effect applied the podcast on midwifery students to obtain knowledge and education on donor eggs.	Design: The quasi-experimental study design.	The mean scores of knowledge pre- and post-intervention podcast groups were greater than the workshop group.
		Participants: Sixty undergraduate midwifery students were s simply randomly allocated to a podcast intervention group and workshop group (*n* = 30 for each group).	
		Intervention: podcast group received three separate 25-min audio files and discussed them in a Telegram-based group.	
Theme 2: To raise comprehension and improve proficient clinical skills			
Clay (37)/United Kingdom	Evaluation of the effectiveness that mobile devices applied whether learners’ learning motivation was increased and enhanced skills in clinical practice	Design: Not explicitly stated.	The results reported mobile learning afforded flexibility in the time and place of learning and captured their interest in the learning material.
		Participants: A convenience sampling recruited eight postgraduate midwives who attended the newborn infant physical examination modules.	
		Intervention: A handheld mobile device (iPod) with several Reusable Learning Objects (RLO) content which was related to newborn infant physical examination to be used in the clinical setting.	
Kardong-Edgren and Emerson (19)/United States	Evaluation of the effectiveness of podcast lectures was used and viewed by students	Design: A descriptive study design	The results showed podcasts of advantages that 82% of students deemed podcasts helped them to understand the subject matter better, 87% aided clarification concepts, and 85% helped to review for homework and examinations.
		Participants: Undergraduate nursing students (*n* = 210)	
		Intervention: Students can listen to the lecture from their computers or download it to an iPod or MP3 player on three courses (pathophysiology, pharmacology, and acute and chronic illness in adults and childbearing)	
McKinney and Page (29)/United Kingdom	An evaluated nursing students’ views on the variety of multimedia resources approach to facilitate the learning of pathophysiology.	Design: Not explicitly stated.	A total 89% of students deemed podcasts or vodcasts to improve their understanding of the pathophysiology. Additionally, using the podcast was convenient and flexible when students moved to other places.
		Participants: Using convenience sampling recruited students (*n* = 125) from the final year of the undergraduate nursing sciences program.	
		Intervention: Making a vodcast or podcast was delivered to iPods, MP3 players, and personal computers in the Applied Biomedical Sciences module course.	
Theme 3: To strengthen self-confidence and improve communication skills			
McSwiggan and Campbell (42)/United Kingdom	To explore students’ experiences of using podcasts for assessment guidance and feedback.	Design: Exploratory qualitative study	The structured, logical approach of assessment guidance podcasts appeared to strengthen self-efficacy by providing readily accessible support and by helping students convert intentions into action.
		Participants: Purposive sampling recruited 18 third-year undergraduate nursing students.	
		Intervention: Applying the self-efficacy theory illuminates students’ use of guidance and feedback podcasts.	
Rogan and San Miguel (41)/Australia	To evaluate an innovation to assist nursing students with English as a second language (ESL) to develop their clinical communication skills and practice readiness by providing online learning resources, using podcast and vodcast technology.	Design: Action research approach	The results showed that the podcast teaching strategy improved their clinical preparation and confidence by increasing their understanding of expectations, clinical language, and communication skills to reach the standards required of nursing graduates and registration authorities.
		Participants: The first-year undergraduate nursing students (*n* = 376), ESL students comprised almost half of each cohort of study participants, primarily from China, Korea, Nepal, and Vietnam, most of whom did not complete high school in English	
		Intervention: The clinical words with 200 audio records with associated images from online were converted to the podcast. Six vodcast scenarios of nurses communicating with patients and staff.	
Theme 4: To provide a useful tool for lecture revision and strengthen supplementary perception			
Meade et al. (28)/United Kingdom	To evaluate both the subjective (student perception) and objective (student use and exam results) usefulness of podcasts of pharmacology lectures.	Design: A cohort study	The results showed that 93% of students used podcasts to revisit a lecture, 85% used podcasts for revision, and 61% used podcasts when they had a specific question. The majority of students deemed podcasts helpful as a learning and revision tool to understand.
		Participants: Graduate nurse non-medical prescribing (*n* = 69)	
		Intervention: Seven key pharmacology lectures were recorded to be divided up into bite-size chunks of lecture material, each containing one or two key concepts, and made available as podcasts to two cohorts of non-medical prescribing students.	

### 3.4. The negative impact of the podcast on nursing education

The disadvantage of podcasting is that students must arrange their study timetables. This leaves the efficiency of their education at the discretion of their motivational or organizational limitations., Similar results were reported that undergraduate and graduate assignments overloaded nursing students to the point that podcast usage decreased (27). In addition, some educators reported that blending podcast use with their course design decreased students’ attendance rate (49), although others reported that students’ class attendance did not decline while using podcasts (48, 50, 51). However, it seems clear that the advantages of the podcast outweigh its disadvantages.

## 4. Discussion

The impact of podcasting on humans cannot be underestimated, especially since remote learning became a standard method during the pandemic. In the past, nursing education has mainly used traditional, lecture-based learning methods, where the teacher is the leader in the classroom through demonstrations or presentations (11), and course content is guided in detail. This situation can lead to students learning to become passive and lacking the ability to problem-solve, think critically, and practice sound judgment (46). Through a synthesis review, we found that nursing course design blended with podcasting showed the following results: that study qualities were low to medium, indicating the studies’ validity as statistical conclusion validity, internal validity, constructive validity, and external validity had to be further confirmed. However, innovative teaching is necessarily integrated into nursing curriculums to bridge the gaps in traditional education.

In Western countries, innovative teaching in nursing has delivered concepts of core nursing such as general clinical skills, communication and teamwork capability, critical thinking, caring, and ethics through podcasts to train nursing students to learn basic nursing core concepts over the past 10 years. Taiwan’s nursing education innovation teaching has become gradually more active. Many educators applied newly-tech learning to teach basic medical and nursing professional knowledge. However, nursing educators seldom use newly-tech learning to teach nursing core concepts. Additionally, podcasting is also rarely applied in nursing education by Taiwan’s nursing educators for the following reasons: podcasts may be lack visual stimulation so which cannot attract students’ concentration, they have a poor internet connection, underestimated ability of podcasting to affect students positively, and insufficient understanding regarding the benefits of podcasting. Nevertheless, research illustrates that podcasting benefits student learning and is a valuable tool to supplement course content. Therefore, podcasts have value and deserve to be promoted in nursing education.

## 5. Conclusions and suggestions

The integration of digital technology has blended with educational teaching strategies to become a trend–podcasting is no longer a new digital technology. Podcasts have already been integrated into nursing education courses in Western countries for a long time. Podcasting has gained attention again with the COVID-19 pandemic. However, there are few podcasts blended into nursing education courses in Taiwan by nursing educators. Based on the above literature review and synthesis, podcasts are more suitable for clinical practice students in nursing education. Repeated listening can help them master clinical professional knowledge and skills. In the future, nursing educators can research whether podcasts can improve nursing students’ knowledge and clinical practice skills. The limitations of this review encompass a limited number of articles that cover approximately 10 years, lacking evidence articles related to the podcast, and no appraisal articles to use the objective instrument.

Considering the wide range of people who access podcasts and the aging population in Taiwan, this should be an especially welcomed trend. Vision declines with age, and others with visual challenges also have reduced visual contact with their surroundings. Hence, efforts to apply the digital technology of podcasting to medical care delivery and the spread of health information will improve society’s physical well-being and increase the external stimulus for people with visual challenges in the future. Furthermore, future research should aim to provide additional evidence regarding the effectiveness of podcast multimedia teaching strategies in nursing education.

## Author contributions

M-CW and J-ST contributed to the conception of the study. J-ST organized and revised the database and references. M-CW analyzed and drafted the manuscript. C-CC revised the manuscript. C-LS supervised the study. Y-PL commented on important views about our revised version. All authors contributed to the article and approved the submitted version.

## Conflict of interest

The authors declare that the research was conducted in the absence of any commercial or financial relationships that could be construed as a potential conflict of interest.

## Publisher’s note

All claims expressed in this article are solely those of the authors and do not necessarily represent those of their affiliated organizations, or those of the publisher, the editors and the reviewers. Any product that may be evaluated in this article, or claim that may be made by its manufacturer, is not guaranteed or endorsed by the publisher.
